# Monitoring the treatment outcome in endometrial cancer patients by CEA and TATI

**DOI:** 10.1007/s13277-016-4784-9

**Published:** 2016-01-16

**Authors:** Barbara Kozakiewicz, Małgorzata Chądzyńska, Ewa Dmoch-Gajzlerska, Małgorzata Stefaniak

**Affiliations:** 1 M.Skłodowska-Curie Memorial Cancer Center and Institute of Oncology, 15 Wawelska St, 02-034 Warsaw, Poland; 2 Institute of Psychiatry and Neurology, Warsaw, Poland; 3 Faculty of Health Sciences in Warsaw, Medical University of Warsaw, Warsaw, Poland

**Keywords:** CEA and TATI in endometrial cancer, ROC curves, Sensitivity and specificity of CEA and TATI

## Abstract

An attempt was made to compare the usefulness of determining markers carcinoembryonic antigen (CEA) and tumor-associated trypsin inhibitor (TATI) in endometrial cancer patients in whom recurrence or distant metastasis was diagnosed in observation after treatment. The study included 316 patients aged 32–81, average age of 61 years, SD = 8.72, with diagnosed endometrial cancer, treated between 1994 and 1995 at the Oncology Center in Warsaw and then under observation from 4 months to 17 years after completion of treatment. The levels of the markers TATI and CEA were assessed from the first five serum samples taken during postoperative radiotherapy and in the initial period of observation after completed treatment. Receiver operating characteristic (ROC) curves were generated, determining the sensitivity and specificity of both CEA and TATI in patients who experienced treatment failure, i.e., recurrence and distant metastasis. Assessing the sensitivity of the marker CEA, it was found that if in the third sample, i.e., during radiation therapy, the marker level increased by more than 20 % compared with the first sample, then recurrence of cancer occurred during the observation period in 75.9 % of patients and metastatic occurred in 69.7 % of patients. In the evaluation of the marker TATI, it was found that if the level of TATI between the first and the third sample increases by 10.6 % from the initial level, then in 84.4 % (sensitivity) of cases, this means the occurrence of cancer recurrence and in 75.7 % (sensitivity) of cases, the occurrence of metastasis. The specificity of both markers is low and not useful diagnostically.

## Introduction

Carcinoembryonic antigen (CEA) was first described in 1956 independently by two groups of researchers—Gold and Freedman as well as Kleist and Burtin. Initially, the antigen was determined in patients with colorectal cancer and was isolated from the intestines of developing fetuses [1, 2]. In 1965, Goldman and Freedman described the relevance of tumor markers and thus initiated the era of discovery of newer and newer markers that coexist with cancer, reproducing the phenomenon of proliferation, differentiation, and death of cancer cells. CEA is considered as one of the markers associated with the stage of tumor development, and in the assessment of the American Society of Clinical Oncology (ASCO), it is the most often studied marker. The diagnostic value of the marker is particularly important in the case of tumors of the colon and rectum [3, 4].

In most cases, high levels of CEA are present in patients with advanced cancer or in the case of multiple metastases [1, 5]. CEA is elevated in 19 % of smokers and 3 % of healthy people and also in pregnant women and alcoholics [6–8].

The first reports of a rise in the level of CEA in patients with endometrial cancer come from the 1970s. In 1977, German scientists Anger and Gleissenberger observed an increase in the concentration of CEA in 38 % of patients with endometrial cancer, which decreased after completion of treatment [9]. In the same period, U.S. researchers have demonstrated a correlation between the increase in the level of CEA with the histological type and the clinical progress of endometrial cancer [10].

Tumor-associated trypsin inhibitor (TATI) is a protein whose concentration in the blood increases in many types of cancers, both benign and malignant. This inhibitor is expressed in the cells of solid tumors and an increase of its level above 20 μg/l in the blood serum, and above 50 μg/l in urine has a negative prognostic significance in the course of ovarian, kidney, bladder, colon, biliary tract, and liver cancer.

TATI was first isolated from the urine of patients with ovarian cancer. It is a protein produced in large amounts by ovarian tumor cells and is included in the group of serine protease inhibitors Kazal-type 1 (SPINK1) [11–15].

Under physiological conditions, TATI inhibits the activity of trypsin, contributing positively to the protection of tissue against its proteolytic effects. However, a negative and synergistic effect of TATI and trypsin is observed in many patients with malignant tumors, contributing to the progress of cancer [16].

It is believed that in cancer patients, the level of TATI increases along with the increase of the level of trypsin. So far, there has not been an explanation of the mechanism of the inhibitor’s action, TATI receptors have not been isolated, but it is suggested that its aggressiveness is modulated by trypsin activity. Thus, TATI expression recognized in the serum and in the cancer tissue is a negative prognostic factor favoring the spread of cancer [12].

In patients with endometrial cancer, there is an increase of the level of TATI in the serum by approximately 21–57 % compared to reference values. An increase of the TATI level by 100 % in the serum occurs in patients with mucinous ovarian cancer [17]. The sensitivity of TATI in patients with endometrial cancer is estimated at approximately 31 % and its specificity at 81 % [18].

The aim of the study is to compare the usefulness of the determination of CEA and TATI in patients with endometrial cancer in whom occurrence of recurrence or distant metastasis of cancer was diagnosed in the course of observation after treatment.

## Material and method

Assessment of the level of CEA and TATI was carried out in 316 patients with endometrial cancer under observation in 1994–1995, who were treated according to staging as determined in accordance with the then applicable FIGO classification from 1988. Subjects’ age ranges from 32 to 81, with average age of 61, SD = 8.72. Observation after treatment in individual patients lasted from 4 months to 17 years. All patients with microscopically diagnosed negative prognostic factors, i.e., deep infiltration of the uterus, poorly differentiated forms of cancer G2 and G3, spread in pelvic area after primary surgery were treated with adjuvant radiotherapy and hormone therapy, followed by post-treatment observation for up to 17 years. Description of demographic variables and clinical status is presented in Tables 1 and 2.

**Table 1:** Descriptive statistics of controlled variables

Variable	Average	Median	Min	Max	Standard deviation
Age	60.38	61.00	32.00	81.00	8.72
Observation time (years)	4.35	2.98	0.24	16.93	3.63
Time free from cancer (years)	3.44	2.26	0.00	16.44	3.60

**Table 2:** Tables of the number of variables describing the clinical status of the patients

	*N*	%
Degree of advancement		
I	51	15.50
II	224	68.09
III	41	12.46
Recurrence		
No	257	78.12
Yes	59	17.93
Meta		
No	248	75.38
Yes	68	20.67
Death		
No	202	61.40
Yes	113	34.35

We analyzed the results of five determinations of both CEA and TATI in serum, which were carried out (every 3–6 weeks) before and after each stage of treatment—brachytherapy and radiotherapy, and during the first three outpatient follow-up visits taking place every 3 months after completion of treatment. Assessment of the marker level was carried out within 5 months from the start of treatment, i.e., surgery.

In the course of the 17-year follow-up period, 59 (18 %) patients were diagnosed with recurrent cancer and 68 (21 %) with distant metastasis. Treatment failure appeared from 6 months to 11 years after completion of treatment.

The values of marker levels in patients with treatment failure ranged from 0 to 344 μg/l for CEA and from 0 to 876 μg/l for TATI.

The paper presents the results of the relationship between the observed levels of markers CEA and TATI and the occurrence of treatment failure (recurrence or distant metastases) compared to a group to patients with successful treatment outcomes. We analyzed the dynamics of changes in marker levels in groups of persons with treatment failure after successful treatment in order to create variables determining level variations in these groups. For marker CEA, the variable “CEA fluctuation in 5 measurements” was created, and for the marker TATI: “average TATI level.” Non-parametric tests were used because of the unfulfilled assumption of a normal distribution of variables tested (Mann-Whitney test for independent groups). Receiver operating characteristic (ROC) curve analysis was also performed.

## Results

Marker CEA level in patients in whom *recurrence of the disease* was determined during observation already in the first sample was significantly different from levels in patients in whom there was no recurrence of cancer (Me = 10 in the group with recurrence, Me = 7 in the group without recurrence, Mann-Whitney *U* test *Z* = −3.094, *p* = 0.002). Full data from all CEA level samples in patients depending on treatment outcome is presented in Table 3.

**Table 3:** Descriptive statistics of CEA level in five determinations, depending on the occurrence of failures in treatment

Subsequent CEA samples		95%CI for Me				Mann-Whitney *U* test^a^	
	Median	−95%CI	95%CI	Min	Max	*Z*	*p*
Total *N* = 316							
C_1	7	3.2	10.8	0	266		
C_2	9	5.6	12.4	0	344		
C_3	10	4.5	15.5	0	251		
C_4	9	2.5	15.5	0	212		
C_5	12	4.5	19.5	2	176		
Recurrence no *N* = 257							
C_1	7	4.8	9.2	0	134		
C_2	8	6.1	9.9	0	112		
C_3	9	5.4	12.6	0	176		
C_4	8	5.4	10.6	0	65		
C_5	10	1.3	18.7	2	176		
Recurrence yes *N* = 59							
C_1	10	0.0	27.7	1	266	−3.094	0.002
C_2	12	0.0	27.5	0	344	−3.251	0.001
C_3	16	0.0	36.8	0	251	−3.816	0.000
C_4	18	0.0	43.9	0	212	−3.342	0.001
C_5	12	3.8	20.2	7	40	−1.310	0.190
Meta no *N* = 248							
C_1	7	4.2	9.8	0	233		
C_2	8	6.0	10.0	0	87		
C_3	9	2.6	15.4	0	251		
C_4	8	0.5	15.5	0	212		
C_5	9	7.3	10.7	2	21		
Meta yes *N* = 69							
C_1	8.5	0.0	22.8	0	266	−1.443	0.149
C_2	12	0.0	25.5	0	344	−3.462	0.001
C_3	14	3.2	24.8	0	176	−3.727	0.000
C_4	17	4.6	29.4	4	142	−4.190	0.000
C_5	20.5	0.0	42.9	3.7	176	−3.429	0.001

^a^Mann-Whitney *U* test was used to compare the results of CEA level samples between groups of cured patients with patients with treatment failure (recurrence or distant metastasis)

**Fig. 1:**
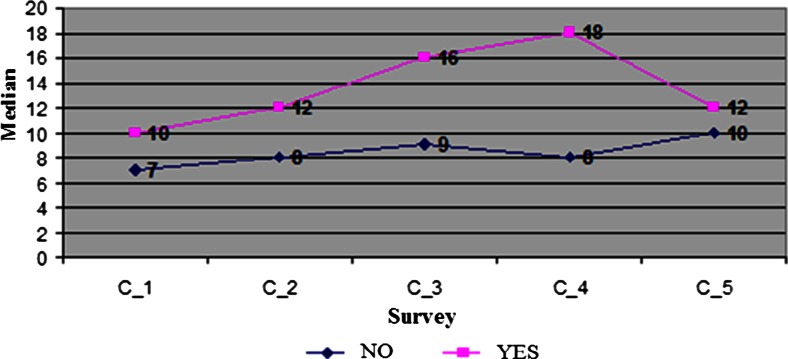
Graph of marker CEA levels in next five subsequent determinations depending on recurrence

The difference between marker CEA levels in groups with and without recurrence was significant until the fourth sample (i.e., the first sample after finishing adjuvant treatment). Only in the fifth sample, i.e., during the follow-up test after completed treatment (approximately 7th–8th month of the study), the levels of markers CEA evened out and there was a significant drop in the level of the marker in the serum of patients with recurrent disease, as shown in the data in Table 3 and graphic chart in Fig. 1. In the same period, the marker level in the group of patients without recurrence was relatively stable: the median within 7–10 μg/l in five samples.

Table 4 shows the descriptive statistics of CEA levels in patients with established disease recurrence and distant metastasis. The calculations use the coefficient “CEA fluctuation in 5 measurements”—which is the difference between the maximum and minimum level of CEA in five subsequent samples.

**Table 4:** Assessment of the fluctuations of CEA levels in five measurements in patients depending on treatment failure

Fluctuation of CEA in 5 measurements			95%CI for Me			
	*N*	Median	−95%CI	95%CI	Min	Max
Recurrence						
No	258	7	3.3	10.7	0	164
Yes	59	14	0.0	33.5	1	261
Meta						
No	248	7.0	2.6	11.4	0	237
Yes	69	15.0	0.0	30.2	0	261

$$ ``\mathrm{C}\mathrm{E}\mathrm{A}\ \mathrm{fluctuation}\ \mathrm{i}\mathrm{n}\ 5\ \mathrm{measurements}" = \mathrm{M}\mathrm{ax}\ {\mathrm{Ci}}_{\left(i=1,\dots 5\right)}\hbox{-}\ \mathrm{M}\mathrm{i}\mathrm{n}\ {\mathrm{Ci}}_{\left(i=1,\dots 5\right)} $$

Assessment of marker CEA showed that in patients with recurrent disease, “CEA marker fluctuation in 5 measurements” is significantly higher than in the group without recurrence (Mann-Whitney *Z* = −4.910085, *p* = 0.0000). In the group without recurrence, the median of this variable is 7 μg/l (CI: (3.3; 10.7)); in the group with recurrence 14 μg/l (CI: (0; 33.5).

Assessing CEA levels in patients with diagnosed *distant metastasis* (meta), significantly greater fluctuations of CEA levels were found in five measurements in the case of metastasis (Mann-Whitney *Z* = −5.47668, *p* = 0.0000). In the group with metastasis, the median of CEA fluctuation in five measurements is 15 μg/l (CI: 0; 30.2) and in the group without metastasis 7 μg/l (CI: (2.6; 11.4).

**Fig. 2:**
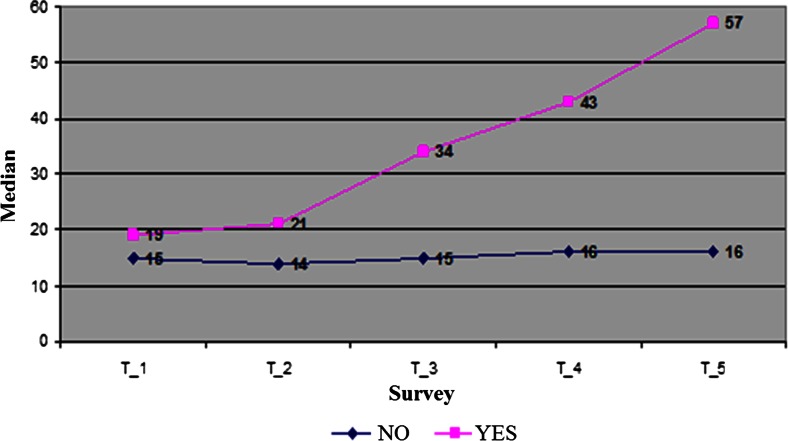
Graph of marker TATI levels in next five subsequent determinations depending on recurrence

Assessing the second marker—TATI, significant differences in the level of the marker was observed in the first five samples between the group with metastatic and the group without metastasis, as well as between the group in which recurrence occurred and the group with no recurrence. Results of TATI levels in patients with recurrence and metastasis are presented in Table 5 and Fig. 2. Therefore, the variable “average TATI level” was created, which is indicative of changes in marker TATI levels in subsequent samples. “Average TATI level” is the average of the first four samples that were taken in the first 12 weeks from the start of the study.

**Table 5:** Descriptive statistics of TATI levels from five samples in patients with treatment failure

Subsequent TATI samples		95%CI for Me				Mann-Whitney *U* test*	
	Median	−95%CI	95%CI	Min	Max	*Z*	*p*
Total *N* = 316							
T_1	16	11.5	20.5	2	302		
T_2	15	10.5	19.5	0	334		
T_3	17	3.9	30.1	2	876		
T_4	17	2.0	32.0	1	543		
T_5	17.5	10.3	24.7	5	87		
Recurrence no *N* = 257							
T_1	15	10.2	19.8	2	302		
T_2	14	10.1	17.9	0	221		
T_3	15	6.5	23.5	2	451		
T_4	16	6.7	25.3	1	329		
T_5	16	10.1	21.9	5	87		
Recurrence yes *N* = 59							
T_1	19	6.8	31.2	7	231	−2.160	0.031
T_2	21	4.8	37.2	0	334	−4.089	0.000
T_3	34	0.0	84.3	5	876	−5.694	0.000
T_4	43	0.0	98.1	7	543	−5.170	0.000
T_5	57	35.0	79.0	6	84	−3.028	0.002
Meta no *N* = 248							
T_1	15	10.1	19.9	2	290		
T_2	14	9.5	18.5	0	334		
T_3	15	0.0	30.4	2	876		
T_4	16	0.0	33.7	1	543		
T_5	15	7.7	22.3	5	87		
Meta yes *N* = 69							
T_1	19	7.7	30.3	7	302	−1.972	0.049
T_2	22	9.5	34.5	0	221	−4.316	0.000
T_3	27	2.5	51.5	5	451	−4.643	0.000
T_4	29	1.4	56.6	10	329	−4.613	0.000
T_5	29.5	15.7	43.3	10	84	−3.941	0.000

Assessing the “Average TATI level” (Table 6) in patients with recurrence, it was found that the average TATI level was significantly higher compared with the cases in which there was no recurrence (Mann-Whitney *Z* = −6.06653, *p* = 0.00000). In cases of recurrence, the median of the “average TATI level” amounts to 28.25 (CI: 11.6; 44.9), and without recurrence, the median is 12 μg/l. (CI: (8.4; 15.6)).

**Table 6:** Assessment of the average level of marker TATI in patients depending on treatment failure

Average TATI level			95%CI for Me			
	*N*	Median	−95%CI	95%CI	Min	Max
Recurrence						
No	257	12	8.4	15.6	2.25	243.5
Yes	59	28.25	11.6	44.9	4	269.5
Meta						
No	248	11.6	6.9	16.4	2.25	269.5
Yes	68	22.1	10.8	33.4	2.25	243.5

Similarly, the groups of patients with known metastasis (META) and without metastasis differ significantly in their average TATI level in the first four assays (Mann-Whitney *Z* = −4.97475, *p* = 0.000001). In patients diagnosed with distant metastasis (Table 5), the average level of TATI in the first four samples was significantly higher, median of 22.1 μg/l (Cl: (10.8; 33.4) compared with patients without metastasis, median of 11.6 μg/l (Cl: (6.9; 16.4).

For TATI, the study of the correlation of dates in the diagnosis of recurrence with the dates of marker determinations showed the existence of a weak statistically significant correlation (*r* = 0.277, *p* = 0.035) of recurrence dates with the dates of the second determination and a very strong correlation (*r* = 0.730, *p* = 0.026) with the dates of the fifth sample (during the first follow-up visit after treatment). This fact may indicate that only few cases of recurrence were diagnosed from the second sample, more often the recurrence was diagnose around the fifth sample. This indicates a relatively late diagnosis of recurrence based on other indicators than TATI.

ROC curve analysis was performed in order to examine the extent to which the observed rapid increase of the levels of markers CEA and TATI between samples determines recurrence and metastasis. Variables were defined for both markers:

$$ {T}_{3-1}=\frac{T_3-{T}_1}{T_1}*100 $$

Where

T3: means the level of marker TATI in the 3rd sample
T1: means the level of marker TATI in the 1st sample

and analogously

$$ {C}_{3-1}=\frac{C_3-{C}_1}{C_1}*100 $$

where

C3: means the level of marker CEA in the 3rd sample
C1: means the level of marker CEA in the 1st sample

**Fig. 3:**
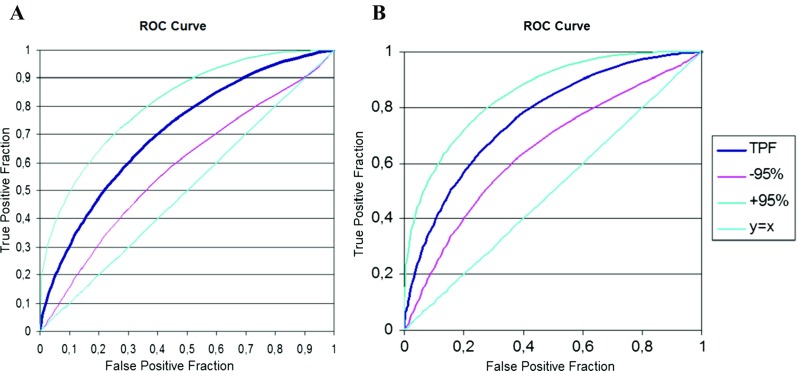
ROC curve with 95 % confidence interval for CEA (**a**) and for TATI (**b**) in the evaluation of recurrence of the disease

The ROC curve, assessing the sensitivity and specificity of CEA and TATI for the prognosis of recurrence, is presented in Fig. 3.

Based on the ROC curve analysis, it was found that if there was an increase in CEA levels by more than 20 % in the third sample compared to the first sample, then cancer recurrence was diagnosed in 75.9 % of cases. Thus, the sensitivity of CEA used to determine recurrence is 76 %. Specificity, or ability to detect patients without recurrence, is 54.3 %, which is quite low. Similarly, in the analysis of ROC curves for evaluation of the occurrence of metastasis based on changes in CEA levels in the third and first sample, it was found that the sensitivity and specificity is low, respectively 69.7 and 53.8 %.

Using the ROC curve analysis (Fig. 3), it was found that if the marker TATI level in the third sample exceeds by 10.6 % the output level, in 84.4 % (sensitivity) of the cases, this means the occurrence of recurrence and in 75.7 % (specificity), the occurrence of metastasis. Marker TATI turns out to be a very sensitive indicator signaling the possibility of recurrence earlier than other traditional methods of diagnosis of recurrence in patients with endometrial cancer.

## Discussion

Endometrial cancer patients are a group of patients with a lack of sufficiently sensitive and specific markers correlated with the diagnosis of the disease and with its course.

CEA is one of the markers investigated most frequently and for the longest period of time in case of patients with cancer in different locations. It was tested in order to determine its usefulness in the early diagnosis of endometrial cancer alongside such markers as SCC (*squamous cell carcinoma antigen*), CYFRA 21-1 (*Cytokeratin 19 fragments*), CA 125 (*cancer antigen*-*125*), CA 19-9 (*cancer antigen*-*19*-*9*), or IAP (*immunosuppressive acidic protein*). However, none of them was recognized by the experts as relevant, i.e., sufficiently sensitive and specific in the diagnosis and prognosis of the course of endometrial cancer.

The increase in the level of the CEA antigen in serum was found in a small group of patients (14–22 %) with endometrial cancer [19]. Bruns et al. observed that it is present only in 6.3 % of patients in the early stage endometrial cancer and not much more often, because in 20 % of patients, in the advanced stages of the disease [20].

The increase in the value of CEA was also used to differentiate adenocarcinoma derived from the uterus and the cervix. Castrillon et al. showed that CEA and vimentin (VIM) are markers that allow to distinguish between cervical cancer and endometrial cancer. Higher values of CEA have been reported more often in patients with cervical cancer, in 62–96 % of cases, and less often in patients with endometrial cancer, in 27–70 % of cases [21, 22].

The highest diagnostic value of CEA was demonstrated for the tumors of the colon and rectum. According to some authors, the increase in CEA levels (>20 ng/ml) is correlated with the degree of advancement of colon cancer and is the higher the greater the progress of the disease [23]. An increasing level of CEA in the blood serum may be associated with the development of cancer and is the first sign of recurrence in about 50 % of patients in whom the tumor was surgically removed [24, 25].

In the recommendations of the American Society of Clinical Oncology (ASCO), National Academy of Clinical Biochemistry (NACB), and the European Group on Tumor Markers (EGTM), determination of CEA is recommended after surgical removal of the tumor in patients with colorectal cancer every 2–3 months for at least 3 years, for the early detection of recurrence or metastasis. However, these recommendations lack the guidelines defining the scope of changes in the level of the marker that would be considered clinically significant. EGTM proposed to consider an increase in the level of the marker in relation to the previous tests of at least 30 % as significant [23, 26].

CEA was also one of the first markers assayed in patients with breast cancer, but now, due to its low sensitivity and specificity, the National Academy of Clinical Biochemistry (NACB) and the European Group on Tumor Markers (EGTM) do not recommend its determination in these patients [27, 28]. However, the build-up of CEA levels is observed in breast cancer patients with more advanced disease, and its elevated values prior to treatment are not considered to be an important prognostic factor [29, 30].

Our study analyzed the value of CEA in patients with endometrial cancer with post-treatment recurrence or metastasis. Evaluation of usefulness of the marker was based on the analysis of its five determinations within 18 weeks of treatment and during the first three follow-up tests after the completion of treatment. Comparing marker levels in cured patients and in patients with current recurrence or metastasis, it was found that CEA is a quite sensitive (75.9 %) indicator for predicting recurrence and a weaker (sensitivity 69.7 %) indicator for predicting the occurrence of metastasis in patients with endometrial cancer. Serial arrays of CEA conducted over a period of 5 months after treatment also showed, in case of colorectal cancer patients, a high sensitivity of 80 % and specificity of 70 % for the early detection of recurrence and metastasis in these patients [31, 32].

In our study, the specificity of CEA for the detection of recurrence and metastasis was low, respectively 54.3 and 53.8 %. The second marker evaluated in patients with endometrial cancer was pancreatic trypsin inhibitor TATI, which is a marker assessed extremely rarely. Originally, its elevated levels were attributed only to diseases of the pancreas and liver. This marker was considered more useful in the diagnosis of bladder cancer than previously identified markers TPA (tissue polypeptide antigen), CEA or SCC (squamous cell carcinoma antigen). TATI levels increase with the progress of bladder cancer in 20–70 % of patients [33, 34].

In patients with prostate, breast, colon, and lung cancer, and even in patients with endometriosis, high sensitivity of TATI assays was observed, which increases with the progress of the disease [35–37].

TATI expression in patients with endometrial cancer with current negative prognostic factors, i.e., lymph node metastasis, infiltration of the cervical canal, or ovarian metastases observed in the histological evaluation after the removal of the reproductive organs, did not differ from the level of the inhibitor in patients in whom these characteristics were not present [38].

In contrast, observation of TATI levels in patients with treatment failures showed that it is more sensitive (84.4 vs 54.3 %) than CEA as an indicator of recurrence of endometrial cancer and a better indicator than CEA (sensitivity 75.7 vs 53.8 %) for the occurrence of distant metastasis [39].

In the light of the study, it appears that the occurrence of recurrence or metastasis may already be suspected in case a significant increase of TATI levels in the first assessment after completed treatment, i.e., in its fourth assessment. If there is an increase in the marker TATI level in the fourth sample by 274 % compared to the first value of its assessment, we can expect cancer recurrence, and in case of a slightly smaller increase of TATI—by 248 %, we should expect distant metastases. Assessment of TATI seems to be a more sensitive indicator of treatment failure than assessment of CEA.

## Conclusions

1. The average of four samples of TATI levels taken within 18 weeks is a sensitive indicator signaling the 84.4 % possibility of recurrence earlier than other traditional methods and a fairly good indicator of 75.7 % chance of distant metastasis.
2. Sensitivity of CEA level fluctuations for the assessment of cancer recurrence and metastasis is low, and amounts to, respectively 75.9 %, and specificity 54.3 % and 69.7 %, specificity 53.8 %.
3. Correlation of the dates of recurrence is indeed very high with the date of the fifth sample, i.e., during the first examination after completion of treatment.
